# Widespread Antibiotic, Biocide, and Metal Resistance in Microbial Communities Inhabiting a Municipal Waste Environment and Anthropogenically Impacted River

**DOI:** 10.1128/mSphere.00346-18

**Published:** 2018-09-26

**Authors:** Aneisha M. Collins-Fairclough, Rebecca Co, Melessa C. Ellis, Laura A. Hug

**Affiliations:** a Faculty of Science and Sport, University of Technology Jamaica, Kingston, Jamaica; b Department of Biology, University of Waterloo, Waterloo, Ontario, Canada; University of Iowa

**Keywords:** antibiotic resistance, biocide resistance, contamination, metagenomics, metal resistance, microbial diversity, municipal solid waste, river

## Abstract

The Riverton City dump is Jamaica’s largest solid waste disposal site, but it lacks engineered protection for leachate containment and treatment. Shotgun metagenomics was used to survey the microbial communities in the Riverton City dump leachate and in surface waters of the Duhaney River, an urban waterway abutting the dump. The community within the leachate pond was taxonomically distinct from that found in the surface waters of the Duhaney River. Higher microbial diversity was observed within the dump leachate, with members of the *Bacteroidetes*, *Firmicutes*, *Gammaproteobacteria*, *Deltaproteobacteria*, and *Tenericutes* being the most abundant, while the river community was dominated by *Alphaproteobacteria*, *Betaproteobacteria*, and *Gammaproteobacteria*. The microbial communities exhibit similar functional potential profiles, including chemoorganoheterotrophy as the dominant metabolism, and the potential to degrade aromatic compounds. From reconstruction of metagenome-assembled genomes (MAGs), organisms within both environments are predicted to survive in the presence of multiple antibiotics, antiseptics, biocides, and metals. Strong virulence potential coincided with the most diverse multiple resistance profiles in 1 of 5 leachate MAGs and 5 of 33 river MAGs. Unexpectedly, the microbial resistance profiles were more varied and widespread in the river populations, where we had expected the chemical composition of the leachate to select and enrich for resistance characteristics. This study provides valuable insights into the total functional potential of a landfill leachate microbial community and identifies possible human health hazards within the Duhaney River and Riverton City dump, urban environments with the potential to impact human populations.

**IMPORTANCE** Landfill leachate is a persistent contamination threat for terrestrial waters. Microbial metabolism in landfills transforms contaminants and contributes to greenhouse gas emissions. A better understanding of landfill-associated microbial communities will inform bioremediation of solid waste environments and improve pathogen monitoring. We leveraged shotgun metagenomics to investigate the microbial communities of the Riverton City dump and the adjoining Duhaney River near Kingston City, Jamaica. We identified no overlap between the microbial communities inhabiting the Riverton City dump leachate and the Duhaney River. Both communities are predicted to degrade aromatic compounds, which are ubiquitous environmental pollutants. Adversely, microbes in both environments are predicted to withstand widely used antibiotics, antiseptics, and metal contamination. The absence of evidence for microbial transfer from the leachate to the river is encouraging; however, the Duhaney River contained several organisms with predicted pathogenic lifestyles, indicating that the river represents a human health risk regardless of impact from the dump.

## Abstract (precis)

Landfill leachate is a persistent contamination threat for terrestrial waters. Microbial metabolism in landfills transforms contaminants and contributes to greenhouse gas emissions. A better understanding of landfill-associated microbial communities will inform bioremediation of solid waste environments and improve pathogen monitoring. We leveraged shotgun metagenomics to investigate the microbial communities of the Riverton City dump and the adjoining Duhaney River near Kingston City, Jamaica. We identified no overlap between the microbial communities inhabiting the Riverton City dump leachate and the Duhaney River. Both communities are predicted to degrade aromatic compounds, which are ubiquitous environmental pollutants. Adversely, microbes in both environments are predicted to withstand widely used antibiotics, antiseptics, and metal contamination. The absence of evidence for microbial transfer from the leachate to the river is encouraging; however, the Duhaney River contained several organisms with predicted pathogenic lifestyles, indicating that the river represents a human health risk regardless of impact from the dump.

## INTRODUCTION

Landfills are among the world’s fastest growing contaminated sites. Municipal landfills are potential contamination sources for surface and drinking water systems, especially when located in urban environments (1, 2). Microbial activity within landfills drives decomposition of inorganic and organic compounds to stabilize the waste. Other microbial activities observed from landfills include ammonium oxidation (3), nitrous oxide reduction (4), and cellulose hydrolysis (5, 6). Despite the importance of these processes and other processes for degradation of landfill waste, the microbial lineages colonizing landfills and their associated metabolic potential are still not well understood. A recent 16S rRNA gene and terminal restriction fragment length polymorphism (T-RFLP)-based survey identified microbial community shifts during refuse decomposition, where distinct populations responded in a successional pattern over time (7). A large-scale survey of 16S rRNA gene-based microbially diverse populations from 19 American landfills identified *Bacteroides*, *Proteobacteria*, and *Firmicutes* as the dominant phyla, with unclassified organisms contributing up to 20% of the surveyed communities (8). The dominance of *Bacteroides*, *Firmicutes*, *Proteobacteria*, or in some cases, *Spirochaetes*, is a common signature from municipal waste sites (8–11), although enrichment culture and bioreactor systems have identified methanogenic *Archaea*, *Thermotogae*, and members of the candidate phylum TM6 as other abundant landfill-associated lineages (12, 13).

To date, attempts to connect microbial metabolisms and resistance profiles with specific populations within municipal waste environments has been limited to microcosm or bioreactor studies (10, 12–14). Wu et al. (14) amended landfill samples with the antibiotic sulfamethazine or oxytetracycline in an effort to decrease denitrification and release ozone-harming NO and N_2_O. While antibiotic use was not clearly correlated with decreased emissions, the authors observed a concomitant increase by up to 10-fold in antibiotic resistance genes (ARGs) in the landfill-derived microbial populations (14). Microcosms amended with cotton identified diverse cellulolytic populations from landfill leachate, including organisms with novel lignocellulose-degrading enzymes with possible biotechnological significance (10). The absence of genomic information for landfill-associated populations has prevented identification of microbial adaptations to the high-metal, high-ammonia, and multiply contaminated landfill environments.

**FIG 1:**
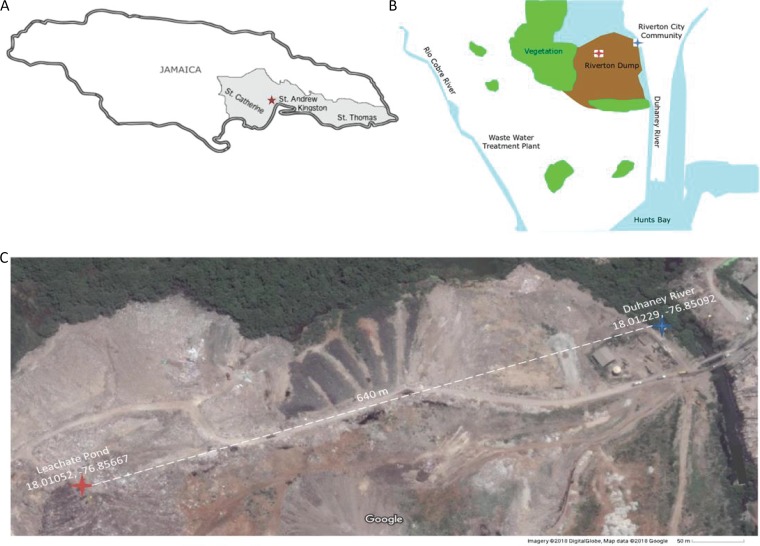
Locations of the Riverton City dump and adjacent Duhaney River sampling sites. (A) Map of Jamaica showing the location of the Riverton City dump (red star) and the parishes it services colored in gray. Kingston City (comprised of Kingston and sections of St. Andrew) is the most populous city served by this dump. (B) Schematic of the Riverton City dump environmental surroundings, including the path of the Duhaney River through vegetation and dump regions. Approximate locations of the leachate and Duhaney River sampling sites are indicated by red and blue stars, respectively. (C) Locations of the leachate pond (red star) and Duhaney River (blue star) sites from which samples were collected and used for metagenome sequencing in this study. Figure 1 was generated in INKSCAPE using maps provided by Google Maps.

Municipal solid waste (MSW) management is particularly important for regions with limited options for site expansion or waste diversion to new locations. This is a specific issue for island nations, including Jamaica. Jamaica generates 800 kilotons of municipal waste per year, with the Riverton City dump, which services Kingston and outlying areas (Fig. 1A), receiving the majority of this waste (15, 16). The Riverton City dump is a low-infrastructure MSW environment, with little to no waste sorting. Large quantities of hazardous waste have historically been deposited at the Riverton City dump, including waste from agricultural, commercial, household, industrial, and medical sources (16). The site has been identified as a major environmental and human health concern, exacerbated by the presence of the Duhaney River running adjacent to the landfill, with direct exposure to the uncontained waste and leachate runoff (Fig. 1B). The Fresh River, which is known to experience sewage contamination from communities (17), converges with the Duhaney River upstream of the Riverton City dump; however, landfill runoff may further negatively impact the Duhaney River. Microorganisms within the dump and potentially within the river are exposed to high concentrations of metals and other toxic compounds.

Here we identify previously unsequenced and uncharacterized microbial populations colonizing the Riverton City dump near Kingston, Jamaica. Metagenome sequencing of samples collected from a leachate pond and the Duhaney River surface waters allowed reconstruction of metagenome-assembled genomes (MAGs) for the most abundant organisms within the leachate and river. From these MAGs, we were able to ascertain the microbial metabolisms present within these anthropogenically impacted environments, including contaminant degradation capacities. We additionally identified virulence signatures and resistance to antibiotics, biocides, and metals to clarify the potential for organisms in these systems to pose a human health hazard.

## RESULTS

### Liquid sampling and analysis.

Surface water samples were taken from a leachate pond at the Riverton City dump and the adjacent Duhaney River near Kingston, Jamaica (Fig. 1). Water samples were sent for analysis to identify key chemical parameters and concentrations of key compounds (Table 1). As expected, the leachate sample showed higher biological oxygen demand (BOD) and chemical oxygen demand (COD), as well as higher metal concentrations compared to the river sample. Compared to Jamaican regulations, the river BOD was the only measurement that exceeded maximum acceptable levels, indicating moderate organic pollution of the Duhaney River at our sampling site (boldface value in Table 1). Leachate metal concentrations, though substantially higher than in the river sample, were well within acceptable standards for sewage and leachate. Metal concentration assays were not sensitive enough to ascertain whether the Duhaney River metal pollution exceeds ecologically safe levels—more-sensitive methods are needed to clarify this point.

**TABLE 1:** Water quality measures for Riverton City dump leachate and Duhaney River surface waters in Jamaica

Parameter (units)^*a*^	Value for parameter in water from:	
	Leachate pond^*b*^	Duhaney River^*c*^
pH	7.3	8.4
BOD (mg/liter)	740	**2.7**
COD (mg/liter)	2.03 × 10^4^	100
Ammonia concn (mg/liter)	N/A	0.46
Phosphate concn (mg/liter)	N/A	0.08
Cadmium concn (mg/liter)	0.059	<0.01
Lead concn (mg/liter)	0.6	<0.02
Mercury concn (mg/liter)	0.003	<0.0001

a BOD, biological oxygen demand; COD, chemical oxygen demand.

b N/A, not available.

c The river BOD that is above the maximum allowable concentration for freshwater systems is shown in boldface type.

### Sequencing.

Total community shotgun sequencing from both the leachate DNA and the river DNA yielded metagenomes of 4.2 and 3.7 GB, respectively. Metagenomes were assembled, and scaffolds longer than 2,500 bp were used for binning to reconstruct metagenome-assembled genomes (MAGs) for the high-abundance populations. The two metagenomes had similar total read numbers and assembly sizes, indicating that the communities were sampled to approximately equivalent depths (Table 2). Using Anvi’o (18), assembled scaffolds were assigned to MAGs, leveraging differential abundance information combined with tetranucleotide frequencies by CONCOCT (19). Differential abundance-based binning was of limited use, as fewer than 0.5% of reads mapped to the nonsource assembly in each case. MAGs were manually refined prioritizing completion and redundancy statistics. After refinement, 13 of 55 leachate MAGs and 3 of 33 river MAGs were high quality (>70% complete and <10% redundant) (Table 3).

**TABLE 2:** Metagenome statistics for the leachate and river samples^*a*^

Sample	No. of reads	Read length (bp)	Total no. of scaffolds; no. of scaffolds of >2,500 bp	*N*_50_ for all scaffolds; *N*_50_ for scaffolds of >2,500 bp	Maximum scaffold length (bp)	% total reads assembled; % reads of >2,500 bp assembled
Leachate	16,673,648	250	555,592; 5,391	12,753; 1,011	532,373	42.7; 16.4
River	14,615,770	250	455,023; 3,348	16,750; 882	511,705	65.2; 24.0

a Good-quality bins were defined as greater than 70% complete and less than 10% redundant. Taxonomic identification was based on a 16 ribosomal protein concatenated gene phylogeny or total gene taxonomic affiliation as calculated by Anvi’o (18). See Table S1 for statistics for all 55 leachate and 33 river MAGs.

**TABLE 3:** Statistics for good-quality MAGs^*a*^

Sample type and MAG	Phylum identification	Closest relative(s)	Identification method	Coverage	% GC	% complete	% redundant	Length (Mbp)	No. of scaffolds	*N*_50_ (kbp)
Leachate										
LB9	*Proteobacteria*	*Desulfococcus* *oleovorans*	RP16 tree	7.4	51.7	96.4	6.7	3.46	198	23.2
LB19	*Bacteroidetes*	LB7, LB17	RP16 tree	10.3	52.9	95.6	3.9	3.26	50	94.6
LB32	*Tenericutes*	LB18_1	RP16 tree	16.5	28.7	95.0	3.6	1.18	52	45.9
LB22	*Firmicutes*	Tepidimicrobium xylanilyticum	RP16 tree	8.1	43.3	94.2	6.2	1.58	137	12.6
LB18_1	*Tenericutes*	LB32	RP16 tree	26.2	33.4	92.1	3.7	1.10	75	18.6
LB10	*Bacteroidetes*	LB12	RP16 tree	8.0	32.8	91.3	3.0	2.31	204	12.2
LB7	*Bacteroidetes*	LB19, LB17	RP16 tree	99.5	42.2	88.5	5.0	2.48	223	12.5
LB27_3	CP CPR2	Uncultivated CPR2 bacteria	RP16 tree	22.2	37.5	88.2	2.2	0.74	8	17.9
LB8	*Proteobacteria*	Desulfotignum phosphitoxidans	RP16 tree	7.9	53.3	85.3	4.2	2.56	207	14.9
LB12	*Bacteroidetes*	LB10	RP16 tree	12.4	43.5	79.8	3.0	2.23	182	15.1
LB26	*Firmicutes*	LB26	RP16 tree	8.2	40.3	79.1	7.8	0.98	102	9.6
LB16	*Firmicutes*	*Clostridiaceae*	RP16 tree	8.9	47.0	71.9	1.9	1.85	152	15.3
LB4_2	*Proteobacteria*	Desulfuromusa kysingii	RP16 tree	77.4	57.6	70.7	0.8	1.13	115	10.6

River										
RB10	*Proteobacteria*	*Loktanella*, RB4_2	RP16 tree	22.4	60.9	86.4	5.4	3.53	14	39.0
RB5	*Proteobacteria*	*Simidula*	RP16 tree	9.3	42.1	85.4	3.6	4.27	177	36.0
RB12	*Proteobacteria*	*Rhodobacter*	Anvi’o	29.6	64.7	71.6	4.2	3.43	33	14.5

a Good-quality bins were defined as greater than 70% complete and less than 10% redundant. Taxonomic identification was based on a 16 ribosomal protein concatenated gene phylogeny or total gene taxonomic affiliation as calculated by Anvi’o (18). See Table S1 for statistics for all 55 leachate and 33 river MAGs.

10.1128/mSphere.00346-18.6 TABLE S1 Summary statistics for all 55 and 33 MAGs reconstructed from the leachate and river metagenomes, respectively. Download Table S1, PDF file, 0.1 MB. Copyright © 2018 Collins-Fairclough et al. 2018 Collins-Fairclough et al. This content is distributed under the terms of the Creative Commons Attribution 4.0 International license.

### Microbial community composition.

**FIG 2:**
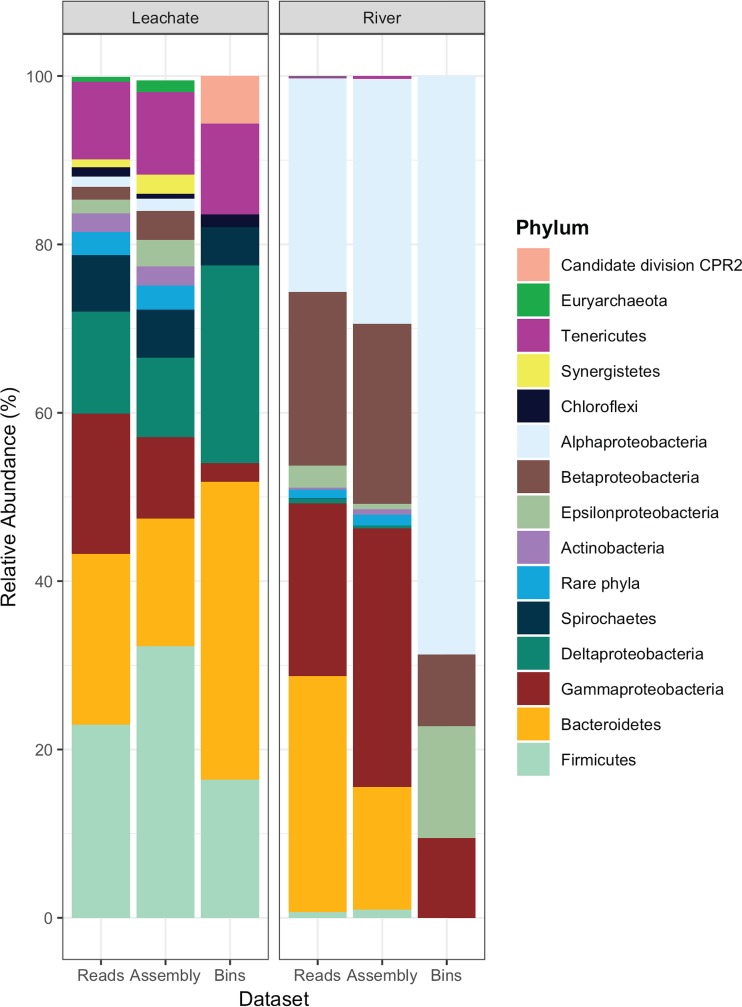
Stacked-bar comparison of relative abundance of organisms from the leachate and river data sets through the sequencing, assembly, and binning pipeline based on marker genes. Phylum-level assignments for unassembled reads, assembled scaffolds, and reconstructed metagenome-assembled genomes (MAGs) are displayed for the leachate and river metagenomes. The abundance affiliated with a particular phylum was calculated as follows: (i) for reads, the percentage of reads affiliated with the phylum out of all identified 16S rRNA gene-containing reads; (ii) for assembled scaffolds, the percentage of scaffolds affiliated with the phylum out of all 16S rRNA gene-encoding scaffolds; (iii) the average fold coverage for scaffolds within the MAG, which was taxonomically classified based on the tree built from concatenated protein alignments of 15 conserved ribosomal proteins. Organisms that occurred at less than 1% were summed together and labeled as rare phyla for clarity of community proportional abundance visualization.

Microbial populations in the leachate and river metagenomes were identified via 16S rRNA genes and/or a set of 15 conserved, single-copy, colocated ribosomal proteins (Table 3; see also Fig. S1 in the supplemental material) (54). The community composition of the reads, assembled scaffolds, and MAGs were compared using predicted 16S rRNA genes to assess whether the binned populations were representative of the total microbially diverse populations sampled from the sites (Fig. 2).

10.1128/mSphere.00346-18.1 FIG S1 Maximum likelihood phylogeny placing the Riverton MAGs on the tree of life. The tree was inferred from a concatenated alignment of 15 ribosomal proteins (RpL2, -3, -4, -5, -6, -14, -16, -18, -22, and -24 and RpS3, -8, -10, -17, and -19), including a reference set of 2,786 publicly available genomes sampled at one genome per genus, 17 leachate MAGs, and 5 river MAGs. Major lineages not containing a MAG of interest have been collapsed. Leachate bins are shown in red, and river bins are shown in blue. Download FIG S1, PDF file, 0.5 MB. Copyright © 2018 Collins-Fairclough et al. 2018 Collins-Fairclough et al. This content is distributed under the terms of the Creative Commons Attribution 4.0 International license.

Reads containing 16S rRNA gene fragments were identified using a hidden Markov model (HMM) search using an HMM built from the SILVA database 16S rRNA genes. A total of 8,762 and 7,731 reads from the leachate and river data sets contained predicted 16S or 18S rRNA gene sequences, of which 7,423 and 6,978 could be taxonomically placed into bacterial or archaeal phyla via BLASTn against the NCBI RefSeq RNA database (Fig. 2).

From the reads, the leachate pond contained three most abundant phyla: *Proteobacteria* (34.6%, with 16.7% *Gammaproteobacteria* and 12.2% *Deltaproteobacteria*, with other classes of *Proteobacteria* occurring at less than 2%), *Firmicutes* (22.9%), and *Bacteroidetes* (20.3%) (Fig. 2). Low-abundance phyla, with relative abundances between 1 and 10%, include the *Tenericutes*, *Spirochaetes*, *Actinobacteria*, and *Chloroflexi* phyla. There were 30 rare phyla occurring at less than 1% abundance in the leachate community. The river sample was dominated by *Proteobacteria*, with 69.7% of 16S rRNA-containing reads classified to that phylum (25.4% *Alphaproteobacteria*, 20.6% *Betaproteobacteria*, 20.6% *Gammaproteobacteria*, 2.6% *Epsilonproteobacteria*, and less than 1% *Deltaproteobacteria*) (Fig. 2). The second most abundant river phylum was *Bacteroidetes* at 28.0%, with the remaining 22 detected phyla occurring at less than 1% of the total community.

From assembled scaffolds, a total of 412 scaffolds from the leachate metagenome and 372 scaffolds from the river metagenome contained predicted 16S rRNA gene sequences (Fig. 2). The microbial composition in the assembled data sets showed that the leachate assembly was dominated by the same major groups as in the reads: 32.3% *Firmicutes*, 15.1% *Bacteroidetes*, and *Proteobacteria*, with 9.7% *Gammaproteobacteria* and 9.4% *Deltaproteobacteria*. *Tenericutes* were also present at 9.7%. In the river assembly, *Proteobacteria* again dominated (82.2%), with 30.7% *Gammaproteobacteria*, 29.1% *Alphaproteobacteria*, and 21.4% *Betaproteobacteria*, with *Epsilonproteobacteria* and *Deltaproteobacteria* at less than 1%. Phyla above 1% abundance in the river also included the *Bacteroidetes* (14.6%) and the *Firmicutes* (1.3%). Many of the identified 16S rRNA genes were present on short scaffolds (∼1 kb), which means that they were not included in the binning process.

**FIG 3:**
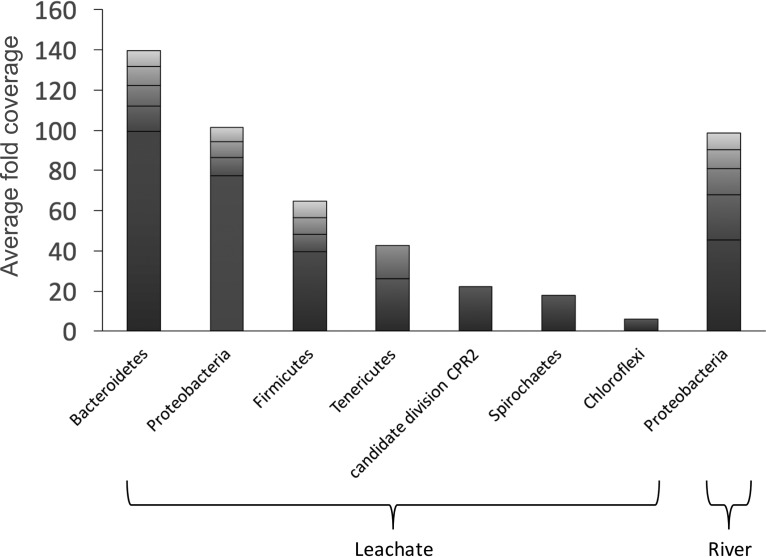
Stacked-bar chart summarizing the microbial community composition of the leachate and river samples based on the MAGs taxonomically placed on the concatenated ribosomal protein tree. Bars represent the summed average fold coverage for each phylum or proteobacterial class. Boxes display individual MAG abundances, colored in a gradient to facilitate distinguishing individual boxes.

The community composition of the MAGs tallied well with the total microbial community composition identified from the reads and assembled scaffolds (Fig. 2), but with significantly lower total diversity. Eighteen MAGs containing the ribosomal protein marker genes from the leachate metagenome were placed into seven phyla. Five MAGs from the river metagenome were able to be classified via ribosomal proteins, with all five identified as *Proteobacteria*. Of the leachate and river MAGs identified via the concatenated ribosomal protein tree, all 13 high-quality leachate MAGs and 2 of 3 high-quality river MAGs were included on the tree (Table 3, Fig. 3, and Fig. S1). From the taxonomically assigned MAGs, the dominant phylum in the leachate community was *Bacteroidetes*, with 35.4% relative abundance across five MAGs. The next most abundant phyla were *Proteobacteria* (4 MAGs, 25.7%, where *Deltaproteobacteria* contributed 23.4%) and *Firmicutes* (4 MAGs, 16.4%). Other phyla within the leachate community were represented by one MAG each, including *Tenericutes*, *Spirochaetes*, *Chloroflexi*, and a member of the candidate phylum CPR2 (20). In contrast, the five MAGs from the river metagenome were all affiliated with the *Proteobacteria*, with *Alphaproteobacteria* dominating (68.7%) over *Betaproteobacteria* (8.5%), *Gammaproteobacteria* (9.6%), and *Epsilonproteobacteria* (13.3%).

Across the reads, assemblies, and MAGs, archaea were a minor proportion of the communities, with their highest abundance at 1.4% in the leachate assembly. Members of the *Crenarchaeota*, *Euryarchaeota*, and *Thaumarchaeota* were present at low abundance in both metagenomes.

### Functional potential.

The functional potential of the metagenomes was determined using the KEGG Metabolic And Physiological potentiaL Evaluator (MAPLE) v. 2.3 (21). Modules with working probabilities (*q* values [false-discovery rates]) below 0.5 at the individual taxon level were considered functional. Modules were categorized according to the MAPLE system, where prokaryotic KEGG modules are considered universal if they are complete in >70% of reference species, restricted if complete in <30% of reference species, and diverse if they show a range of completion across reference species. The designation of rare was additionally applied to modules if they are complete in <10% of reference genomes (22). We note that pathways predicted to be absent from either or both samples may be present in the low-abundance members of the leachate and river microbial communities, whose genomic potential was not captured by our sequencing depth.

In a global functional analysis using each metagenome as a single data set, the leachate and river metagenomes had 183 and 200 modules that were predicted to be functional, with 142 of these modules shared between the two metagenomes. The shared KEGG modules belong to universal (24%), restricted (20%), and diverse (53%) categories, distributed across pathways, complexes, transporters, and functional sets. Shared KEGG pathway modules include energy metabolism such as carbon fixation and dissimilatory nitrate reduction (KEGG database accession no. M00530), as well as modules for assimilatory sulfate reduction (M00176) and metabolism of amino acids, carbohydrates, lipids, and nucleotides (Fig. S2). Shared KEGG complex modules include ribosomes, DNA and RNA polymerase complexes, complexes for ATP synthesis, and transport complexes for minerals, organic ions, lipids, saccharides, peptides, and nickel (Fig. S3). Some shared modules are rare in their distribution among prokaryotes, including pathways for polyamine biosynthesis (M00134), vitamin and cofactor biosynthesis (M00124 and M00121), drug efflux (M00720), as well as transport systems for biotin (M00581), capsular polysaccharide (M00249), rhamnose (M00220), and l-arabinose or lactose (M00199). These rare modules have been shown to contribute to stress tolerance, viability, or virulence in some microbes (23–25). Benzoate degradation (M00551) is a restricted, rare pathway that is also shared by MAGs of the leachate and river metagenomes. Functional set modules that are shared between the river and leachate metagenomes include aminoacyl tRNA (M00360), two-component regulatory systems (TCRS) for carbon metabolism (M00475), nitrogen regulation (M00498), surface appendages (M00501 and M00515), stress responses (secretion [M00448] and phosphate starvation [M00434]), redox response (M00523), and transport of magnesium (M00444) and potassium (M00454). Functional sets for multidrug efflux (M00647) and aminoglycoside resistance through protease FtsH (M00742) are also shared (Fig. S4).

10.1128/mSphere.00346-18.2 FIG S2 Venn diagram depicting shared functions between the microbial communities of the Riverton City dump leachate and the Duhaney River based on the presence of MAPLE-predicted KEGG pathway modules. Shared modules are located in the intersection of both ellipses, while modules exclusive to each environment are listed outside the intersection but within the ellipse bearing the label of the environment in which it occurs. The MAPLE classification of the modules based on their distribution among bacteria is reflected in the color highlighting the module name as follows: white, universal modules; gray, diverse modules; black, restricted modules. Asterisks indicate rare modules. Download FIG S2, TIF file, 0.5 MB. Copyright © 2018 Collins-Fairclough et al. 2018 Collins-Fairclough et al. This content is distributed under the terms of the Creative Commons Attribution 4.0 International license.

10.1128/mSphere.00346-18.3 FIG S3 Venn diagram depicting shared functions between the microbial communities of the Riverton City dump leachate and the Duhaney River based on the presence of MAPLE-predicted KEGG complex modules. Shared modules are located in the intersection of both ellipses, while modules exclusive to each environment are listed outside the intersection but within the ellipse bearing the label of the environment in which it occurs. The MAPLE classification of the modules based on their distribution among bacteria is reflected in the color highlighting the module name as follows: white, universal modules; gray, diverse modules; black, restricted modules. Asterisks indicate rare modules. Download FIG S3, TIF file, 0.6 MB. Copyright © 2018 Collins-Fairclough et al. 2018 Collins-Fairclough et al. This content is distributed under the terms of the Creative Commons Attribution 4.0 International license.

10.1128/mSphere.00346-18.4 FIG S4 Venn diagram depicting shared functions between the microbial communities of the Riverton City dump leachate and the Duhaney River based on the presence of MAPLE-predicted KEGG function modules. Shared modules are located in the intersection of both ellipses, while modules exclusive to each environment are listed outside the intersection but within the ellipse bearing the label of the environment in which it occurs. The MAPLE classification of the modules based on their distribution among bacteria is reflected in the color highlighting the module name as follows: white, universal modules; gray, diverse modules; black, restricted modules. Asterisks indicate rare modules. Download FIG S4, TIF file, 0.6 MB. Copyright © 2018 Collins-Fairclough et al. 2018 Collins-Fairclough et al. This content is distributed under the terms of the Creative Commons Attribution 4.0 International license.

No universally distributed modules were exclusive to the leachate or river metagenome. Rather, the functional capacities of the microbial communities found in the river and leachate pond were distinguished by modules that are restricted, diverse, and in some cases, rare (Fig. S2 to S5). Pathways specific to the leachate metagenome included the reductive acetyl coenzyme A (acetyl-CoA) pathway (M00377), formaldehyde assimilation (M00345), and dissimilatory sulfate assimilation (M00596), all of which are anaerobic processes. The benzoyl-CoA degradation pathway (M00541) for degradation of aromatic compounds was present in the leachate metagenome, but not in the river metagenome, while the aromatic-degrading catechol *meta*-cleavage pathway (M00569) was specific to the river metagenome, along with the homoprocatechuate pathway for aromatic amino acid degradation (M00533). The river metagenome contains organisms carrying genes encoding components involved in the Entner-Doudoroff pathway, assimilatory nitrogen reduction (M00531), denitrification (M00529), and thiosulfate oxidation via the sulfur oxidation (SOX) pathway (M00595). Multiple transport module complexes differentiated the leachate and river microbial communities (Fig. S3). Transport systems for fluoroquinolone (M00224) and the insecticide gamma-hexachlorohexane (lindane [M00669]) are present in the leachate community, but not the river community. The river community contain genes that encode 19 exclusive functional set modules compared to 8 exclusive functional set modules for the leachate community (Fig. S4). The leachate-exclusive functional sets include an additional phosphate starvation TCRS (SenX3-RegX3 [M00443]), TCRS for metal tolerance (M00499), amino sugar metabolism (M00502), and glutamine utilization (M00518). Functional set modules exclusively identified in the river data set include those for cationic antimicrobial peptide resistance (M00727 and M00728), TCRS for trimethylamine oxide (TMAO) respiration (M00455), nitrate respiration (M00471), copper tolerance (M00452), quorum sensing (M00453), alginate production (M00493), chemotaxis (M00506), chemosensing (M00507), as well as response to osmotic (M00445) and envelope (M00447 and M00450) stress. Signature modules provide a higher-level characterization of communities. Both the leachate and river metagenomes encoded the signature module for nitrate assimilation (M00615) but were distinguished by the acetogen signature (M00618), present only in the leachate community, and by signatures for sulfate-sulfur assimilation (M00616), imipenem (M00745), and multidrug resistance (M00649), which were present only in the river community (Fig. S5).

10.1128/mSphere.00346-18.5 FIG S5 Venn diagram depicting shared functions between the microbial communities of the Riverton City dump leachate and the Duhaney River based on the presence of MAPLE-predicted KEGG signature modules. Shared modules are located in the intersection of both ellipses, while modules exclusive to each environment are listed outside the intersection but within the ellipse bearing the label of the environment in which it occurs. The MAPLE classification of the modules based on their distribution among bacteria is reflected in the color highlighting the module name as follows: white, universal modules; gray, diverse modules; black, restricted modules. Asterisks indicate rare modules. Download FIG S5, TIF file, 0.3 MB. Copyright © 2018 Collins-Fairclough et al. 2018 Collins-Fairclough et al. This content is distributed under the terms of the Creative Commons Attribution 4.0 International license.

### Resistance profiles.

Based on the MAPLE total community analyses, microbial resistance to stress, metals, and antibiotics emerged as distinguishing features for these two environments. As both sample types came from anthropogenically contaminated sites, we were interested in which organisms were adapted to the nonpristine conditions and what mechanisms were involved in this adaptation. To that end, the resistance profile for each MAG was determined for biocides, antibiotics, and metals using DeepARG (26) to identify potential antimicrobial resistance genes, and BLASTp searches against the BacMet database of biocide and metal resistance proteins (27). As an additional important consideration, we examined the virulence potential for these MAGs using the virulence factor database (VFDB [28]) to determine which, if any, organisms at the Riverton City dump or the Duhaney River are predicted pathogens.

### Distribution of metal resistance.

**FIG 4:**
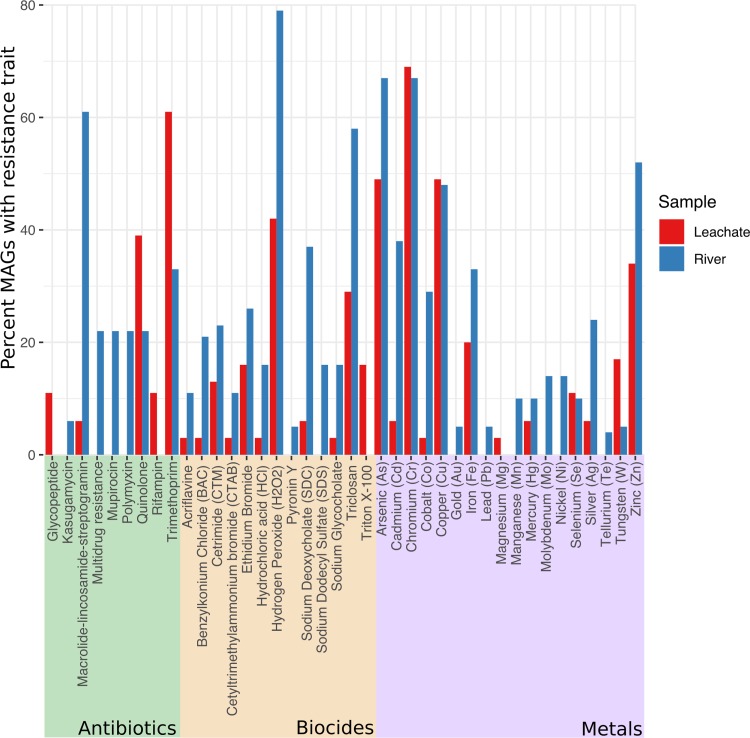
Bar chart depicting percentage of MAGs with predicted resistance to antibiotics, biocides, or metals within the leachate and river metagenomes, with resistance categorized by the class of compound. Antibiotic resistance was predicted using the DeepARG tool, while biocide and metal resistance profiles were predicted by BLASTp searches against the BacMet experimental database.

The capacity for metal resistance was predicted for 64% of MAGs in both the leachate (35/55) and river (21/33) communities, with resistance to multiple metals predicted for 45% of leachate MAGs (25/55) and 42% of river (14/33) MAGs. Ninety-one metal resistance proteins were predicted from both metagenomes combined, where only 26% of these proteins were shared. The most frequently predicted metal resistances were to arsenic, chromium, zinc, and copper for both the leachate and river samples (Fig. 4). Cadmium and cobalt resistance were more than six times more frequent in river MAGs than in leachate MAGs, while resistance to silver was present in three times as many river MAGs than leachate MAGs. The occurrence of tungsten resistance is three times more frequent in the leachate than the river. The river community has a greater diversity of predicted metal resistance compared to the leachate community, with resistance to gold, lead, manganese, molybdenum, and tellurium exclusively found in the river MAGs. The broadest spectra of predicted metal resistance for leachate community members were observed in two *Deltaproteobacteria*, members of the *Desulfobacteraceae* (leachate bin 8 [LB8] and LB9), with resistance to six different metals each. Gammaproteobacterial MAGs predicted to be *Pseudomonadaceae* (LB2_2 and LB2_3) as well as one unclassified MAG (LB6) each encoded predicted resistance to five different metals. In the river MAGs, two *Gammaproteobacteria* (*Pseudomonadaceae* river bin 2_2 [RB2_2] and RB5) had the broadest metal resistance spectra, with predicted resistance to nine different metals, followed closely by two *Alphaproteobacteria* (*Rhodobacterales* RB10 and RB12) and one unclassified MAG (RB7) with predicted resistance to eight metals.

### Distribution of biocide resistance.

We used BLAST to search the BacMet experimental database to identify predicted proteins conferring resistance to biocides. Biocide resistance genes were predicted in 56% (31/55) and 58% (19/33) of Riverton City dump leachate and Duhaney River MAGs, respectively (Fig. 4). Only 24% (10/42) of biocide resistance categories were shared by both communities. The leachate and river communities shared a highest frequency of biocide resistance to hydrogen peroxide and triclosan. Similar to the metal resistance analysis, the river community demonstrated a stronger biocide resistance profile than the leachate community. Resistance to acriflavine, benzalkonium chloride, cetyl trimethylammonium bromide (CTAB), hydrochloric acid, sodium deoxycholate, and sodium glycocholate was more than three times more frequent among the river MAGs compared to the leachate MAGs. Resistance to Triton X-100 was predicted for the leachate community only, while sodium dodecyl sulfate (SDS) resistance and pyronin resistance were predicted for the river community only.

### Distribution of antibiotic resistance.

From the DeepARG predictions, antibiotic resistance was detected in 33% of leachate MAGs (18/55) and 55% of river MAGs (18/33). A total of 13 antibiotic resistance proteins were identified from the leachate and river metagenomes combined, and only 15% of these occurred in both metagenomes. The river MAGs contained a greater diversity of antimicrobial resistance genes (ARGs), with resistance to seven antibiotic classes predicted compared to five for the leachate MAGs (Fig. 4). Of the MAGs containing predicted ARGs, trimethoprim resistance was most common in the leachate MAGs (11/18 [61%]), followed by resistance to quinolone (7/18 [39%]). Resistance to glycopeptides and rifampin was predicted for two MAGs, while one leachate MAG is predicted to have resistance to the macrolide class of antibiotics. In contrast, macrolide resistance was the highest occurring ARG class within the river MAGs (11/18 [61%]), followed by resistance to trimethoprim (6/18 [33%]), and then resistance to mupirocin, polymyxin, and quinolone, each of which was predicted in 22% of river MAGs (4/18). Kasugamycin resistance was seen only in the river community (1/18), while glycopeptide resistance and rifampin resistance were predicted only in leachate MAGs. Complementing DeepARG, MAPLE also predicted multiple multidrug efflux or resistance pumps as well as protease FtsH aminoglycoside resistance in both metagenomes. KEGG modules inferring resistance to imipenem and cationic antimicrobial peptides were exclusive to the river metagenome, while the fluoroquinolone transport system was predicted as functional in the leachate metagenome only. Multidrug resistance was predicted for three leachate MAGs taxonomically placed as *Gammaproteobacteria* (*Halomonadaceae* LB11), *Bacteroidetes* (LB12), and *Firmicutes* (*Clostridiaceae* LB16). Five river MAGs had predicted multidrug resistance: one *Gammaproteobacteria* (*Pseudomonadaceae* RB2_2) resistant to six antibiotic classes, one unclassified MAG (RB2_3) and one *Gammaproteobacteria* (*Pseudomonadaceae* RB5) resistant to five antibiotic classes, one unclassified MAG (RB2_7) resistant to four antibiotic classes, and one *Alphaproteobacteria* (*Rhodobacterales* RB10) resistant to two antibiotic classes.

### Distribution of virulence.

**FIG 5:**
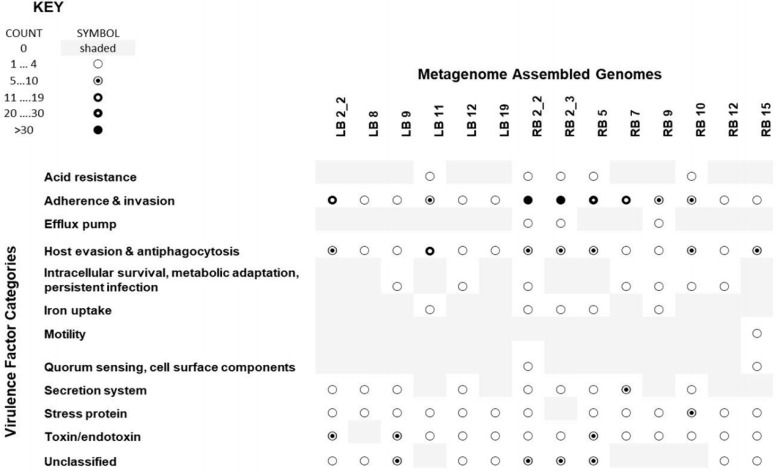
Dot plot depicting the distribution of virulence factor categories among river and leachate MAGs that are predicted pathogens. The virulence factor categories are derived from the keywords assigned to individual virulence factors in the Virulence Factor Database (28).

The majority of MAGs from both the leachate (40/55 [73%]) and river (27/33 [82%]) metagenomes contained at least one virulence factor. Distinct virulence genes were grouped on the basis of their functional identification (ID) within the VFDB (28). The presence of homologues to 10 or more distinct virulence factors was taken as stronger evidence of a likely pathogenic phenotype. Using this criterion, six leachate MAGs and eight river MAGs were identified as predicted virulent populations. All 14 MAGs possessed genes involved in adherence and invasion, host evasion, and antiphagocytosis (Fig. 5).

Genes encoding likely stress proteins and toxins were present in 13 of the 14 MAGs predicted to be virulent. Five MAGs possessed homologues of the Helicobacter pylori acid resistance urease complex (29). None of the five were classified as *Helicobacter* or even as *Epsilonproteobacteria* (LB5 [unclassified], RB2_2 [*Pseudomonadaceae*], RB2_3 [unclassified], RB5 [*Pseudomonadaceae*], and RB10 [*Rhodobacterales*]). BacMet predicted that these same five MAGs exclusively possess homologues to RpoS, a sigma factor that promotes survival of cells exposed to extreme acid (31). Unclassified MAG RB2_3 additionally carries a gene encoding a homologue of the EefA protein, a component of the tripartite efflux system that promotes the survival of Klebsiella pneumoniae in inorganic acid environments (30). These multiple pieces of evidence strongly suggest that these five populations (represented by MAGs LB11, RB2_2, RB2_3, RB5, and RB10) may be able to establish gastrointestinal infections. These MAGs are also multidrug and multimetal resistant, making them of particular interest for human health considerations.

## DISCUSSION

In this study, we used shotgun metagenomics to examine the taxonomic composition, functional potential, and resistance profiles of the microbial communities occupying a leachate pond at the Riverton City dump and the adjacent Duhaney River in Jamaica. Both sites are anthropogenically impacted, the Riverton City dump by deposited refuse, and the Duhaney River from sewage inputs in the Fresh River that converges with it (17). Surface waters were sampled in both cases, with genome-resolved metagenomics providing insight into the dominant organisms inhabiting these contaminated sites. From this survey, there is no evidence for the sampled leachate pond contaminating the Duhaney River site, which is approximately 600 m away. Water quality measures do not indicate cross-contamination of these systems. No MAGs were observed in common between the two sites, and reads cross-mapped at extremely low levels to their nonsource metagenomes. Temporal sampling, including at a location where leachate runoff might directly enter the Duhaney River, will be required to assess whether the dump leachate does impact the river. Our results suggest that the Duhaney River microbial populations are adapted to high metal concentrations and exposure to antibiotics—the source of these stressors is not yet known.

The microbial communities identified from the two sites are dominated by specific, nonoverlapping groups. The leachate pond community exhibited higher microbial diversity than the river community did and had more major divisions represented at high abundance (>1%), including *Bacteroidetes*, *Firmicutes*, *Proteobacteria* (*Gammaproteobacteria* and *Deltaproteobacteria*), and *Tenericutes*, whereas the river community was dominated by *Proteobacteria* (*Alphaproteobacteria*, *Betaproteobacteria*, and *Gammaproteobacteria*). The phylum-level diversity of the microbial communities as identified from 16S rRNA gene-containing reads is reasonably well matched by the high-quality MAGs, though with a significant reduction in the number of organisms represented. The 16S rRNA genes identified from scaffolds indicate hundreds of different populations are present in these environments. Our MAGs represent the most abundant populations from the phyla identified in the leachate pond and river samples. The only major lineage lost during the binning process was the *Bacteroidetes* in the river sample. Upon closer examination, several poor-quality (<70% complete) MAGs associated with the *Bacteroidetes* group *Flavobacteria* (RB1_2, RB 1_3, and RB1_4) were reconstructed, indicating the presence of strain variation that may have hindered MAG resolution. There is evidence for novel microbially diverse populations within the dump environment—one MAG was taxonomically assigned to the Candidate Phylum Radiation candidate phylum CPR2, a radiation currently represented by four environmentally derived genomes, including the MAG from this study (20) (Fig. S1). The low abundance of archaeal 16S rRNA genes in the reads and assembled data sets alongside the absence of any archaeal MAGs was unexpected, but it may be typical of solid waste environments. A survey of landfill leachate across American landfills using 16S rRNA amplicon sequencing identified *Archaea* at a relative abundance of 0.8 to 4.35% in most landfills, with only two sites hosting slightly higher abundances, with *Euryarchaeota* at 6.28 and 9.33% (8).

The two sites shared more similarities on a functional level compared to taxonomic affiliations, although the functional similarities were largely underlain by different mechanisms (shared resistance proteins were 26% for metals, 24% for biocides, and 15% for antibiotics). This minority overlap of functional mechanisms is consistent with the differential taxonomy observed between the two metagenomes. Universal KEGG modules were, as expected, represented in both samples. Rare pathways shared by both sites included functions implicated in cell viability and stress tolerance as well as virulence. The microbial communities of both the leachate pond and the neighboring Duhaney River contained microbes with the predicted aerobic pathway to degrade aromatic hydrocarbons, common terrestrial contaminants which are widely present in manufactured products. Degradative metabolic capabilities of this kind are beneficial for limiting environmental accumulation of hazardous compounds, and the predicted presence of this function at a municipal solid waste site is particularly advantageous. Functional differences between the two sites included signatures for anaerobic lifestyles, metal tolerance profiles, biofilm formation capacity, osmotic stresses, and multidrug resistance.

The metagenomes demonstrated remarkable resistance profiles, with some MAGs showing the genetic potential to survive in an environment containing multiple antibiotics, biocides, and several metals. Jamaican soils have heavy metal concentrations that exceed world averages (32), and combined with anthropogenic pollutants (domestic and otherwise), could drive selection of these multiply resistant microbes. For both sites, the predicted resistance mechanisms for biocides and metals are predominantly extrusion based, using efflux pumps, rather than biotransformation of the compounds. The prevalence of efflux-based resistance mechanisms implies that, while these organisms are capable of propagating and persisting within these contaminated environments, their ability to withstand the contaminants is likely not connected to metabolizing or otherwise transforming these compounds, at least using known mechanisms found in databases. To our knowledge, there is no current overview of the prevalence of antibiotic resistance genes across the genomes of organisms in the environment. Our data suggest that these mechanisms can be widespread for anthropogenically impacted sites, with one third to one half of MAGs containing at least one ARG.

The metagenomes provide evidence of microbes with the potential to establish infection and survive in the presence of multiple antibiotics. Six of the potentially most virulent MAGs also had the most extensive predicted resistance profiles, with three or more homologues to distinct antibiotic classes, biocides, and metals (Table 4). The occurrence of these potentially virulent and resistant microbes in the Duhaney River, a lotic body, indicates greater opportunity for their dissemination compared to the stagnant leachate ponds in the dump. Since these metagenomes were generated from samples collected at a single time point over a limited area, more comprehensive studies could further illuminate the environmental and health impact of the Riverton City dump and the Duhaney River. Nevertheless, the findings generated here justify a review of management of human access to both the Riverton City dump and the Duhaney River to safeguard public health.

**TABLE 4:** Profile of the six MAGs with the most extensive predicted resistance and virulence phenotypes^*a*^

Parameter or resistance category [compound(s) or metal]^*b*^	Value for parameter or no. of resistance genes in MAG					
	LB11	RB2_2	RB2_3	RB5	RB7	RB10
% genome completion	40	44	37	85	32	86

Antibiotic						
Aminoglycoside^+^	1	1	1	1	1	1
CAMP^+^ and polymyxin	0	2	2	1	1	1
Imipenem^+^	0	0	1	0	1	0
Kasugamycin	0	1	0	0	0	0
Macrolide	0	0	1	1	1	1
Multidrug*	3	4	3	2	2	1
Mupirocin	0	1	0	1	1	0
Quinolone	1	1	1	1	0	0
Trimethoprim	1	1	0	1	1	0

Biocide						
Acriflavine	0	0	1	0	0	0
Ethidium bromide	1	1	2	1	0	1
Hydrochloric acid	1	1	2	1	0	0
Hydrogen peroxide	1	0	1	1	2	1
QAC	1	1	1	2	1	2
Sodium deoxycholate	1	3	2	0	1	0
Sodium dodecyl sulfate	0	1	0	0	0	0
Triclosan	1	2	1	3	2	1

Metal						
Arsenic	2	1	3	3	2	2
Cadmium	0	0	0	1	2	1
Chromium	4	3	4	3	2	3
Cobalt	0	1	1	1	0	0
Copper	0	3	2	1	1	2
Gold	0	0	0	0	1	0
Iron	1	1	1	0	1	3
Manganese	0	0	0	0	0	4
Mercury	0	0	0	3	0	0
Molybdenum	0	0	0	0	0	1
Nickel	0	1	0	3	0	0
Selenium	1	1	0	1	0	0
Silver	0	1	0	0	1	0
Zinc	0	3	3	3	1	2

a These MAGs have the genetic potential for resistance to at least three classes of antibiotics, biocides, and metals, in addition to possessing 10 or more different virulence factors. Antibiotic resistance was predicted primarily by DeepARG, with additional annotations from MAPLE KEGG. Biocide and metal resistance were predicted by BLASTp search of the BacMet experimental database, and virulence was predicted by BLASTp search against the VFDB.

b Antibiotic resistance was predicted primarily by DeepARG, with additional annotations from MAPLE KEGG (indicated by a + superscript). Predictions by both DeepARG and MAPLE KEGG are indicated by an asterisk. Abbreviations: CAMP, cationic antimicrobial peptide; QAC, quaternary ammonium compound.

## MATERIALS AND METHODS

### Sample collection.

Two locations in Jamaica were selected for sampling based on accessibility—a leachate pond that passively develops within the Riverton City dump and a site in the Duhaney River adjacent to the dump, approximately 600 m from the leachate pond. There is no obvious fluid flow between the two sampling sites. The two locations were chosen as representative sites for the two environments.

Leachate samples were collected from the periphery of a perennial leachate pond at the Riverton City dump (18.01052 N, 76.85667 W) in Jamaica. An autoclaved disposable jug was first rinsed with a surface sample from the leachate pond. The jug was used to scoop leachate from the top 1-m layer of one edge of the pond into two autoclaved 2.5-liter conical flasks. Water samples were collected from the Duhaney River in Jamaica, which passes through the Riverton City dump (18.012292 N, 76.850922 W). Autoclaved conical flasks were rinsed with surface water from the river. The flasks were then dipped in the river and filled to two-thirds capacity with surface water from the periphery of the river. Flasks were sealed with Parafilm M and capped with aluminum foil prior to transportation to the laboratory. All samples were transported directly to the laboratory and stored at 4°C, and DNA was extracted within 4 days of sample collection.

### Water testing.

Aliquots from both the leachate and river samples were sent for water quality testing to measure pH, biological oxygen demand (BOD), and chemical oxygen demand (COD), and total concentrations of ammonia, phosphate, cadmium, lead, and mercury. All analyses were conducted by the Scientific Research Council’s Analytical Services department (Jamaica). Results were compared to the Jamaican Natural Resources Conservation Authority’s published regulations to determine whether measured values exceeded legislated maximum levels.

### DNA extraction and sequencing.

Leachate samples (approximately 100 ml) were filtered using six 0.22-µm polyethersulfone membranes (diameter, 47 mm; Sterilitech), switching filters upon clogging. DNA was extracted from the membranes using the MoBio PowerWater DNA isolation kit according to the manufacturer’s instructions. In parallel, the MoBio PowerSoil DNA isolation kit was used to extract DNA from 18 ml of leachate as follows. First, 1.0-ml aliquots of leachate samples were centrifuged at 14,000 rpm for 30 min. Supernatants from the tubes were transferred to new tubes. The supernatant and pellet from each tube were processed separately for DNA extraction using the MoBio PowerSoil DNA isolation kit, according to the manufacturer’s instructions, with the supernatant and pellet as input in place of soil. The resulting eluates from all leachate DNA extractions were pooled and purified using a phenol-chloroform extraction.

The Duhaney River water samples were filtered through a total of nine 0.22-µm polyethersulfone membranes using a vacuum pump attached to a Buchner funnel. The filtrate was further filtered through seven 0.03-µm polyethersulfone filters. Approximately 10 liters of river water was filtered in total. Filters were used for DNA extraction using the MoBio PowerWater DNA extraction kit. DNA eluates from all river extractions were pooled for sequencing.

Shotgun metagenomic sequencing was conducted by the McMaster University Farncombe Metagenomics Facility in Canada. Prior to sequencing, the pooled leachate DNA was further purified using AMPure beads. The NEBNext Ultra DNA kit was used for library preparation from 100 ng from each DNA sample. The leachate library and the river library were pooled, and a sequencing library was prepared using the Illumina MiSeq 250-bp paired-end read v2 kit.

### Metagenomic pipeline.

Metagenomes were assembled and annotated as described previously (33). Briefly, reads from both metagenomes were quality trimmed with Sickle (34). Paired-end reads were assembled using IDBA-UD (35) under default parameters, with each metagenome assembled separately. Open reading frames (ORFs) were predicted using MetaProdigal (36), and annotated via USEARCH (37) against KEGG (38), UniRef90 (39), and InterproScan (40) databases. Annotations were ranked A to E and reported as follows: (A) reciprocal best hits with bit score of >350, then (B) reciprocal best hit to UniRef with a bit score of >350, (C) best hit to KEGG with a bit score of >60 or best hit to UniRef90 with a bit score of >60. Proteins with InterproScan matches but no other hits were ranked as (D), and hypothetical proteins that were predicted open reading frames but no further annotations were ranked (E). Reads from both leachate and river were then mapped to both leachate and river assemblies using Bowtie 2 v. 2.2.6 (41) to determine contig coverage statistics and enable abundance-based binning metrics.

Anvi’o v. 2.0.2 (18) was used to bin the scaffolds, manually refine the bins, and visualize the data. First, a contig database was created from the respective metagenome’s contig file, and open reading frames were identified through Prodigal v. 2.6.2. These ORFs were used solely for assessing bin completion, while MetaProdigal annotations described above were used for metabolic reconstructions and phylogenetic inferences. Genes corresponding to single-copy core gene bacterial (19, 42–44) and archaeal (45) gene collections were identified using HMMER v. 3.1b2 (46). Coverage information for each contig was determined via samtools (47). Contigs were binned using CONCOCT (19), leveraging nucleotide frequency information as well as differential coverage. All programs were used under default parameters as implemented by Anvi’o. The bins were manually refined based on completion and redundancy statistics in the Anvi’o interactive interface. A bin was considered a high-quality MAG if it was greater than 70% complete and had less than 10% contamination.

### Phylogenetic inference.

Organism taxonomic placement was inferred from a phylogenetic tree built from concatenated protein alignments of 15 conserved, single-copy ribosomal proteins (RpL2, -3, -4, -5, -6, -14, -16, -18, -22, and -24, and RpS3, -8, -10, -17, and -19). MAGs containing more than 50% of these marker genes were included in the phylogenetic inference alongside a reference set comprising one member of each genus for which sequenced genomes are available (from reference 55). Each protein data set was aligned individually using MUSCLE v 3.8.31 (48), and then the 15 alignments were concatenated. Alignments were edited using Geneious v. 10.0.5 (49). Alignment positions with greater than 95% gaps were removed, and C and N termini with nonconserved regions were trimmed. Taxa with information for less than 50% of the trimmed concatenated alignment were removed. The final concatenated alignment contained 2,786 sequences and 2,470 amino acid positions. A maximum likelihood tree was constructed using RAxML-HPC v. 8.2.10 (50) on the public web server CIPRES Science Gateway v. 3.3 (51) using the LG+gamma protein substitution matrix and with automatic bootstopping to determine the optimal number of bootstrap replicates. The phylogenetic tree was visualized in FigTree v. 1.4.3 (http://tree.bio.ed.ac.uk/software/figtree/). All software programs used were operated under default parameters unless otherwise stated.

### 16S rRNA gene community profiles.

We used the SILVA database core alignment to search for 16S rRNA genes within our data sets. The SILVA alignment contained 592,605 bacterial and 25,026 archaeal 16S rRNA genes. Reads with best hits to eukaryotes were removed from analyses from this point onwards. A hidden Markov model (HMM) was built using HMMER 3.1b2 for the nonredundant small-subunit reference data set (nonredundant at 99% identity) from the SILVA 132 release (52). The 16S rRNA gene HMM was searched against the trimmed leachate and river reads using the per-target output, with an E value of 1e^−5^. Reads identified from this search were BLASTn searched against the RefSeq RNA database (release 87) (53) and the NCBI taxonomy database (November 2017). Top hits with a minimum E value of 1e^−40^ were used to identify taxonomy at the phylum level for the 16S rRNA gene-containing reads. For the assembled data, the same 16S rRNA gene HMM was searched against the leachate and river assembled scaffolds using the same pipeline and parameters as for the read search.

### Functional potential assessment.

The functional potentials of the assembled leachate and river metagenomes were compared using the Metabolic And Physiological potentiaL Evaluator (MAPLE) v 2.3 (21). Modules with working probabilities (*q* values [false-discovery rates]) below 0.5 at the individual taxon level were considered functional. The metagenomes were further explored for their potential for resistance to antibiotics, metals, and biocides, as well as virulence. Assembled river and leachate metagenomes were used as input in a BLASTp search against the BacMet database of experimentally validated genes for resistance to metals and biocides (27). Antimicrobial resistance was assessed using the DeepARG tool (26). The pathogenicity of the recovered genomes was assessed by BLASTp search of the experimentally confirmed Virulence Factor Database (VFDB) (28). Functional predictions were accepted when homologues showed amino acid identities greater than or equal to 50% over >70 amino acids. Functional predictions were accepted for DeepARG and BLASTp hits that shared identities of 50% or more to database homologues over an alignment length of at least 70 amino acids.

### Data availability.

Riverton City dump leachate files are deposited under BioProject PRJNA475763 and biosample SAMN09401598. Leachate reads are deposited to the SRA under accession no. SRR7299214. The Duhaney River files are deposited under BioProject PRJNA475764 and biosample SAMN09401599. River reads are deposited to the SRA under accession no. SRR7346984.
