# Supplementary material for: Widespread Antibiotic, Biocide, and Metal Resistance in Microbial Communities Inhabiting a Municipal Waste Environment and Anthropogenically Impacted River
Source: mSphere. 2018 Sep 26;3(5):e00346-18. doi: 10.1128/mSphere.00346-18 (PMC6158514; doi:10.1128/mSphere.00346-18)
Supplement: TABLE S1 [file sph005182642st1.pdf]

Table S1: Summary statistics for all 55 and 33 MAGs reconstructed from the leachate and river metagenomes, respectively.

	Phylum identification	Closest relative	Means of ID	Coverage	% GC	% Complete	% Redundant	Length (Mbp)	# scaffolds	N50 (Kbp)
**LEACHATE**										
**LB9**	Proteobacteria	*Desulfococcus oleovorans* Hxd3	RP16 tree	7.4	51.7	96.4	6.7	3.5	198	23.2
**LB19**	Bacteroidetes	LB7, LB17	RP16 tree	10.3	52.9	95.6	3.9	3.3	50	94.1
**LB32**	Tenericutes	LB18_1	RP16 tree	16.5	28.7	95.0	3.6	1.2	52	45.9
**LB22**	Firmicutes	*Tepidimicrobium xylanilyticum*	RP16 tree	8.1	43.3	94.2	6.2	1.6	137	12.6
**LB18_1**	Tenericutes	LB32	RP16 tree	26.2	33.4	92.1	3.6	1.1	75	18.6
**LB10**	Bacteroidetes	LB12	RP16 tree	8.0	32.8	91.3	3.0	2.3	204	12.2
**LB7**	Bacteroidetes	LB19, LB17	RP16 tree	99.5	42.2	88.5	5.0	2.5	223	12.6
**LB27_3**	candidate division CPR2	Uncultivated CPR2 bacteria	RP16 tree	22.2	37.5	88.2	2.2	0.7	8	179.3
**LB8**	Proteobacteria	*Desulfotignum phosphitoxidans*	RP16 tree	7.9	53.3	85.3	4.2	2.6	207	14.9
**LB12**	Bacteroidetes	LB10	RP16 tree	12.4	43.5	79.8	2.9	2.2	182	15.1
**LB26**	Firmicutes	Firmicutes, LB26	RP16 tree	8.2	40.3	79.1	7.8	1.0	102	9.6
**LB16**	Firmicutes	Clostridiaceae	RP16 tree	8.9	47.0	71.9	1.9	1.9	152	15.3
**LB4_2**	Proteobacteria	*Desulfuromusa kysingii* DSM 7343	RP16 tree	77.4	57.6	70.7	0.8	1.1	115	10.6
LB17	Bacteroidetes	LB7, LB19	RP16 tree	9.5	41.4	66.5	2.7	1.2	157	8.1
LB21	None			28.3	49.9	66.5	5.6	2.1	128	22.2
LB25	None			5.1	42.9	62.3	2.1	0.9	109	8.9
LB27_2	None			21.0	32.4	60.4	0.9	0.3	30	11.2
LB29_2	None			22.9	24.7	57.2	4.4	0.6	54	12.3
LB23	None			9.8	48.9	57.1	3.5	1.8	125	17.6
LB33	Chloroflexi	Dehalococcoidete*s*	RP16 tree	6.0	44.0	50.2	0.6	0.4	59	6.8
LB18_3	Firmicutes	*Erysipelothrix rhusiopathiae*	RP16 tree	39.5	33.9	49.7	0.9	0.6	55	11.3

LB20	None			8.1	31.6	48.0	9.0	1.5	144	11.9
LB2_2	Proteobacteria	*Pseudomonas*	Anvi'o	14.2	57.5	47.3	3.5	2.0	221	9.6
LB24	None			5.0	40.1	46.9	13.3	0.7	109	6.4
LB3_2	None			4.9	36.1	46.0	9.9	1.1	150	6.7
LB15	Proteobacteria	*Halomonas elongata* DSM 2581	RP16 tree	8.9	57.5	41.2	2.9	1.2	158	7.1
LB11	Proteobacteria	*Halomonas*	Anvi'o	15.8	67.6	40.2	5.0	2.7	191	18.0
LB13_3	None			12.9	45.7	39.4	0.0	0.3	23	18.8
LB1_4	None			5.2	66.6	37.7	0.4	0.9	109	7.5
LB34	None			9.8	45.1	35.8	0.0	0.3	42	7.0
LB6	None			5.0	46.6	35.3	2.2	1.8	245	7.1
LB31	None			6.8	47.2	32.1	0.8	0.6	80	7.4
LB29_3	None			8.1	26.2	31.1	1.9	0.3	29	9.6
LB13_2	None			5.5	46.5	31.0	0.9	1.2	160	7.3
LB3_3	None			8.8	35.8	27.6	3.7	0.8	88	9.0
LB4_3	Spirochaetes	Spirochaeta sp. Buddy	RP16 tree	18.0	52.0	26.4	0.2	1.6	154	10.6
LB14	None			4.4	48.5	24.9	3.1	1.1	167	6.5
LB2_3	Proteobacteria	*Pseudomonas*	Anvi'o	15.6	59.1	20.4	2.8	0.7	91	7.5
LB1_3	None			10.4	66.9	19.6	0.0	0.3	23	12.0
LB3_4	None			3.7	40.6	18.3	0.0	0.1	10	6.6
LB1_2_1	None			8.6	68.7	17.9	2.0	0.8	82	10.5
LB36	Proteobacteria	*Pseudomonas*	Anvi'o	20.3	50.5	12.8	6.5	0.1	7	10.8
LB29_4	None			4.5	25.7	11.1	0.0	0.0	5	5.6
LB30	None			6.2	33.9	10.8	0.0	0.6	88	7.5
LB18_2	None			9.4	32.8	9.3	0.0	0.2	25	7.9
LB28	None			4.4	55.3	9.2	1.2	0.6	93	6.4
LB5	None			10.2	36.7	5.3	1.7	2.1	246	9.0
LB3_5	None			30.6	38.5	0.4	0.0	0.4	40	9.1
LB35	None			18.6	49.4	0.0	0.0	0.2	24	7.5
LB37	None			13.5	41.9	0.0	0.0	0.0	4	6.2

LB38	None			5.7	44.0	0.0	0.0	0.0	3	5.6
LB39	None			46.6	62.2	0.0	0.0	0.0	3	8.2
LB40	None			9.9	52.2	0.0	0.0	0.0	1	35.5
LB41	None			5.6	41.2	0.0	0.0	0.0	1	7.4
LB42	None			7.2	41.1	0.0	0.0	0.0	1	7.0

	**Phylum identification**	**Closest relative**	**Means of ID**	**Coverage**	**% GC**	**% Complete**	**% Redundant**	**Length (Mbp)**	**# scaffolds**	**N50 (Kbp)**
**RIVER**										
**RB10**	Proteobacteria	*Loktanella*, RB4_2	RP16 tree	22.4	60.9	86.4	5.4	3.5	14	390.4
**RB5**	Proteobacteria	*Simidula*	RP16 tree	9.3	42.1	85.4	3.6	4.3	177	36.0
**RB12**	Proteobacteria	*Rhodobacter*	Anvi'o	29.6	64.7	71.6	4.2	3.4	33	145.3
RB8_2	Proteobacteria	*Hydrogenophaga*	RP16 tree	8.4	53.3	68.6	3.2	0.8	99	8.3
RB3	None			13.2	65.5	51.0	6.6	2.0	268	7.5
RB15	Proteobacteria	*Arcobacter*	Anvi'o	9.0	31.6	50.6	1.9	1.0	102	11.5
RB13	Proteobacteria	*Arcobacter*	RP16 tree	13.1	37.4	45.3	8.8	1.4	130	10.1
RB2_2	Proteobacteria	*Pseudomonas*	Anvi'o	18.6	55.6	44.0	2.6	3.6	198	23.3
RB9	None			6.2	55.0	42.0	3.7	1.5	191	7.9
RB6	None			7.5	66.6	41.2	2.9	1.6	212	6.9
RB4_2	Proteobacteria	*Loktanella*, RB10	RP16 tree	45.5	59.6	39.0	2.0	1.4	159	7.5
RB14	None			11.7	45.0	38.9	2.2	1.5	134	13.2
RB2_3	None			10.5	56.8	37.3	2.3	3.5	260	16.2
RB7	None			7.2	52.4	31.8	3.5	2.1	201	11.6
RB4_3	None			32.6	62.9	28.7	0.0	0.4	19	33.8
RB1_2	Bacteroidetes	*Flavobacterium*	Anvi'o	17.4	31.8	28.5	10.3	2.4	183	16.0
RB1_3	Bacteroidetes	*Flavobacterium*	Anvi'o	29.6	31.5	26.5	4.6	2.0	177	11.5

RB8_3	None			14.7	52.1	23.3	0.4	0.6	41	35.8
RB11	None			22.8	57.7	20.1	9.6	1.5	168	8.6
RB1_4	Bacteroidetes	*Flavobacterium*	Anvi'o	52.5	31.6	17.2	2.3	1.4	164	8.3
RB8_4	None			13.6	53.3	16.5	0.0	0.6	29	131.4
RB4_4	None			30.3	59.9	14.4	0.0	1.0	54	25.9
RB1_5	None			10.1	31.2	11.6	0.9	1.2	120	10.0
RB17	None			5.2	42.5	5.3	0.6	0.3	53	6.0
RB21	None			8.6	59.3	5.3	0.2	0.0	5	6.1
RB16	None			8.9	30.0	4.8	0.0	0.9	54	22.1
RB20	None			5.7	66.3	3.9	0.0	0.1	13	6.2
RB18	None			118.6	41.2	0.2	0.5	0.6	46	14.6
RB19	None			74.7	62.2	0.0	0.0	0.3	11	28.2
RB1_6	None			5.9	30.7	0.0	0.0	0.1	22	6.5
RB22	None			74.0	42.9	0.0	0.0	0.0	5	11.6
RB23	None			8.2	25.1	0.0	0.0	0.0	1	7.1
RB2_4	None			59.1	54.3	0.0	0.0	0.0	5	5.7
