# Supplementary figures and images for: Widespread Antibiotic, Biocide, and Metal Resistance in Microbial Communities Inhabiting a Municipal Waste Environment and Anthropogenically Impacted River
Source: mSphere. 2018 Sep 26;3(5):e00346-18. doi: 10.1128/mSphere.00346-18 (PMC6158514; doi:10.1128/mSphere.00346-18)

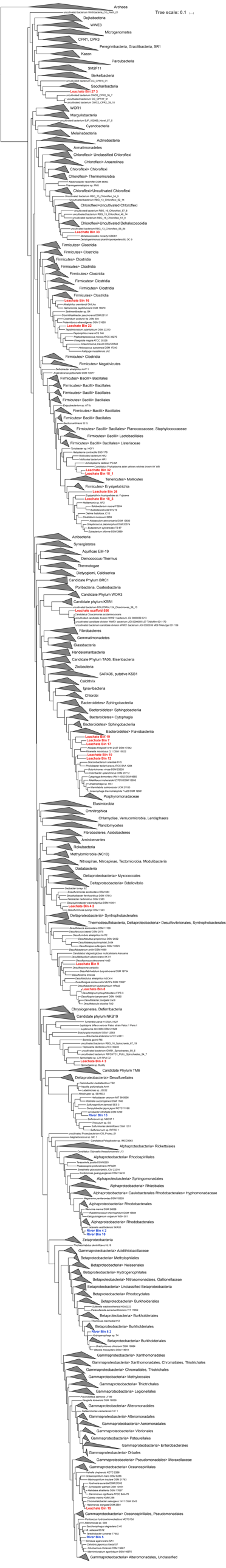

Supplement: FIG S1 [file sph005182642sf1.pdf]

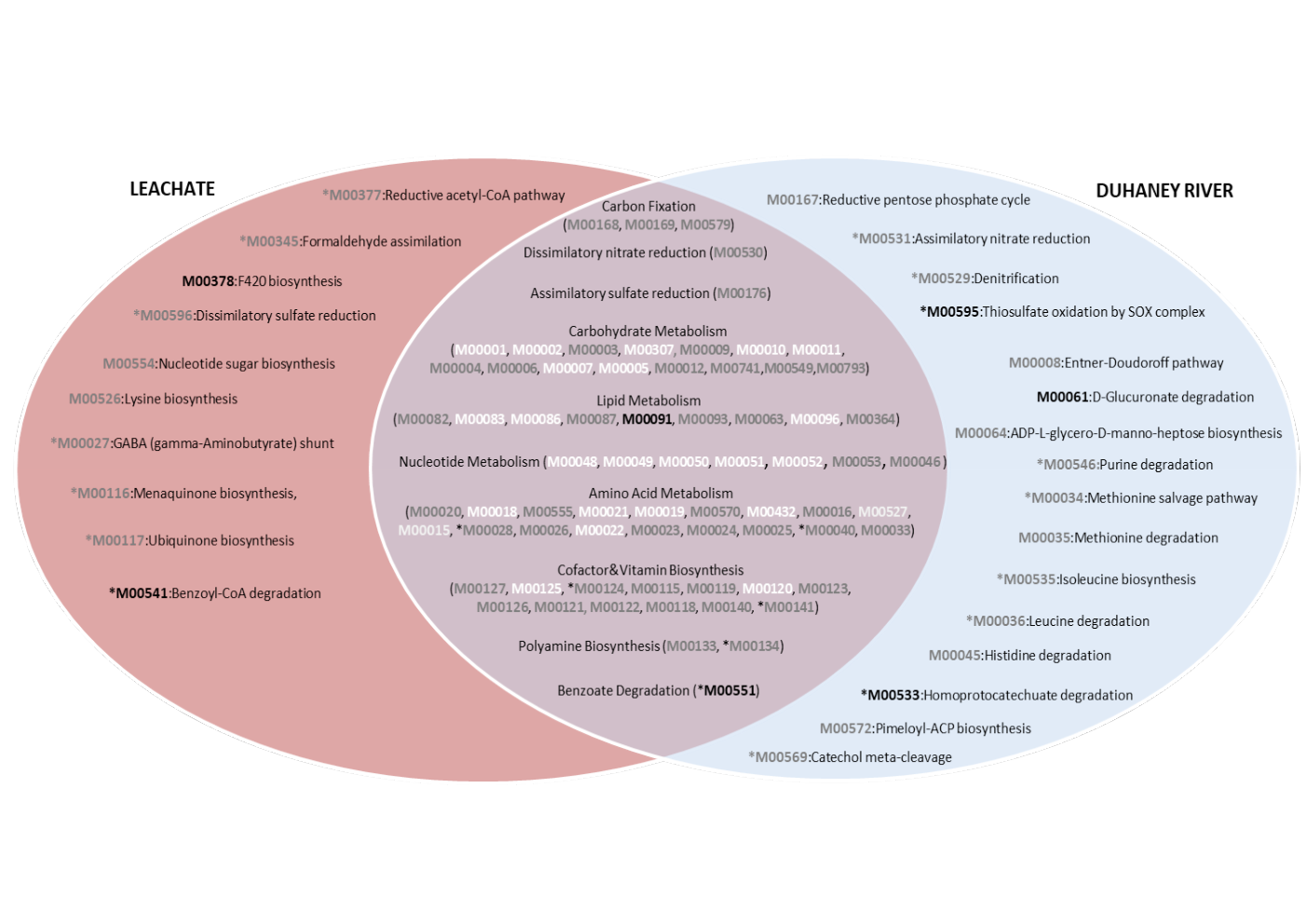

Supplement: FIG S2 [file sph005182642sf2.tif]

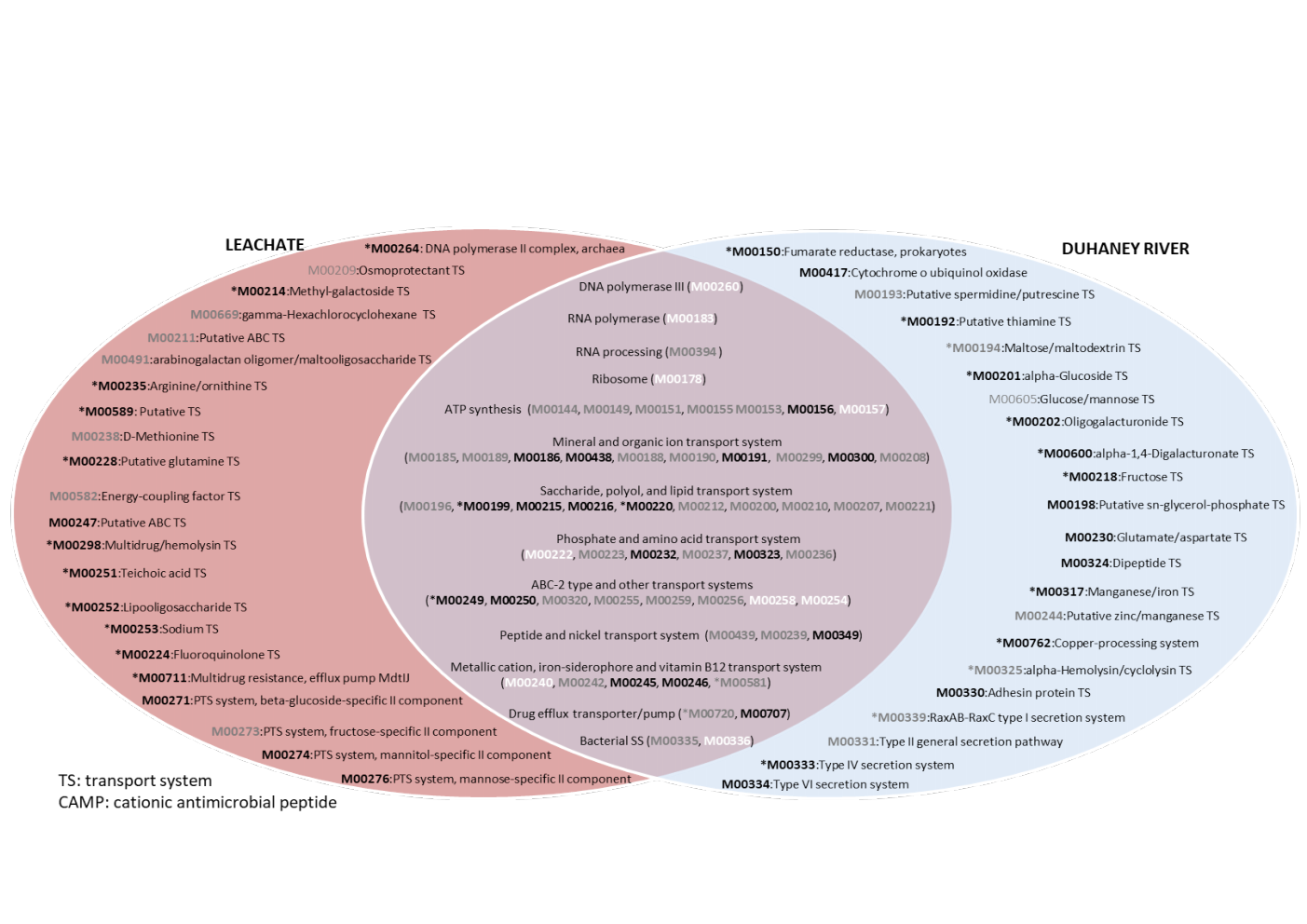

Supplement: FIG S3 [file sph005182642sf3.tif]

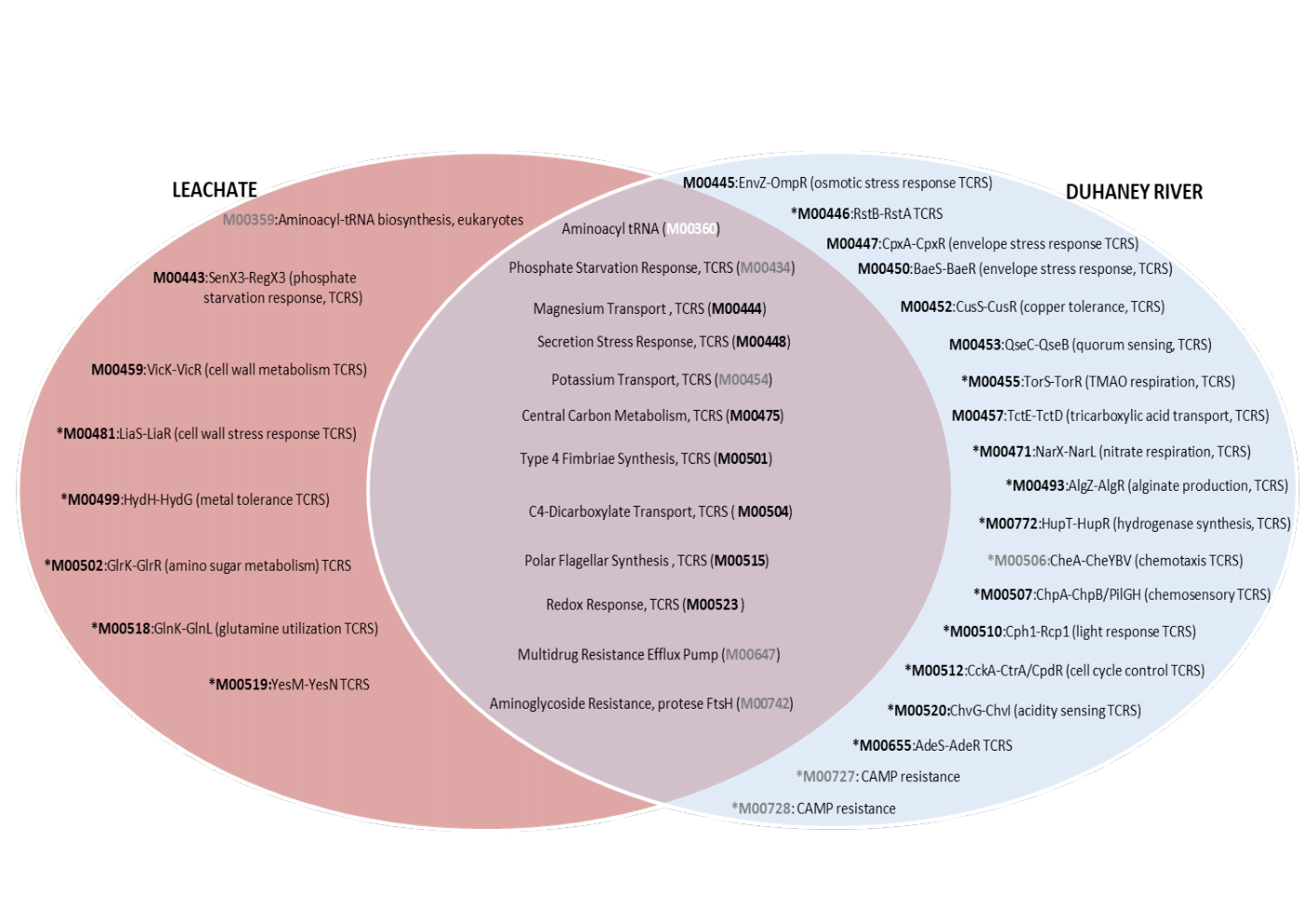

Supplement: FIG S4 [file sph005182642sf4.tif]

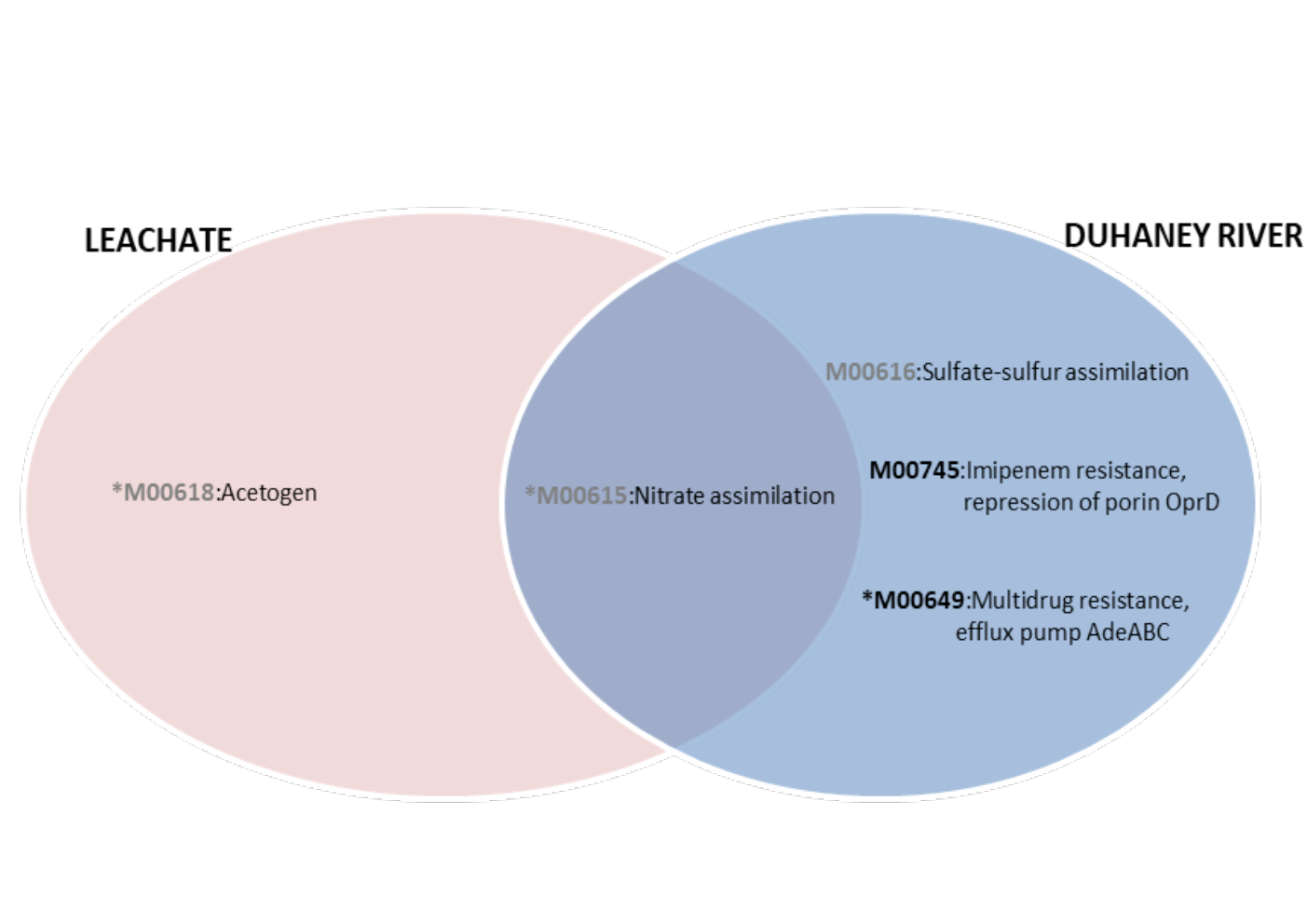

Supplement: FIG S5 [file sph005182642sf5.tif]
